# The Editor's Letter-Box

**Published:** 1912-05-04

**Authors:** 


*To the Editor of* The Hospital.

Sir,—I am not partial to writing anonymous letters, nor to thrusting myself uninvited into other people's quarrels. It is with some diffidence, therefore, that I once more ask to trespass upon your space in connection with the dispute between Messrs. Hall and Snell on the one side, and yourself and your contributors on the other.

It seems to me that the real *gravamen* of the original criticisms upon the premiated plans is in some danger of being missed, while the parties wrangle over the exact significance of an ill-worded sentence in the conditions of the competition. Neither Mr. Hall nor your F.R.I.B.A.s in their answer to him (see The Hospital, April 27, pp. 109, 110) offer any remarks upon the definite statement made in the original article (The Hospital, March 2, p. 564) that three of the roads had been designed with gradients respectively of 1 in 3, 1 in 4.7, and 1 in 5. There are other definite criticisms in the same section of the article; but I select this in particular because the point is one which a layman like myself can comprehend. What I should like to know is whether those roads have the gradients alleged. If they have, the roads are impracticable and the design premiated by Mr. Hall must stand condemned. If they have not, you and your contributors owe Mr. Hall an apology.

The essence of the controversy is, after all, whether the plan selected by Mr. Hall is the best of those sent in. Your contributors say not, and it is for them and for you to prove the statement. They criticised in detail several features of the plan; and many of those criticisms have not been answered. What outsiders interested in hospital affairs want to get at is whether the criticisms were fair or not. So far we have not seen any attempt to refute them; but we are loth to see judgment go by default in this way, for there are two sides to every case. Will not the successful architect or the assessor (Mr. Hall) offer some defence on these points?—Yours faithfully,

April 27. Hospital Medical Officer.

[Note.—We have had to hold over till next week an "Engineer's" reply to Mr. Kay's question in our last issue on "A Suction Gas Plant *v.* Boiler Engine."]

